# An Interesting Case of Intramuscular Myxoma with Scapular Bone Lysis

**DOI:** 10.1155/2017/1690409

**Published:** 2017-01-17

**Authors:** Jérôme Tirefort, Frank C. Kolo, Alexandre Lädermann

**Affiliations:** ^1^Division of Orthopaedics and Trauma Surgery, Department of Surgery, Geneva University Hospitals, Geneva, Switzerland; ^2^Rive Droite Radiology Center, Geneva, Switzerland; ^3^Faculty of Medicine, University of Geneva, Geneva, Switzerland; ^4^Division of Orthopaedics and Trauma Surgery, La Tour Hospital, Geneva, Switzerland

## Abstract

*Introduction.* Intramuscular myxoma is a rare benign primitive tumor of the mesenchyme founded at the skeletal muscle level; it presents itself like an unpainful, slow-growing mass. Myxomas with bone lysis are even more rare; only 7 cases have been reported in the English literature, but never at the shoulder level.* Case Presentation.* We describe an 83-year-old patient with a growing mass in the deltoid muscle with unique scapular lysis, without any symptom. Magnetic resonance imaging (MRI) and a biopsy were performed and the diagnosis of intramuscular myxoma has been retained. In front of this diagnosis of nonmalignant lesion, the decision of a simple follow-up was taken. One year after this decision, the patient was still asymptomatic.* Conclusion.* In the presence of an intramuscular growing mass with associated bone lysis, intramuscular myxoma as well as malignant tumor should be evoked. MRI has to be part of the initial radiologic appraisal but biopsy is essential to confirm the diagnosis. By consensus, the standard treatment is surgical excision but conservative treatment with simple follow-up can be an option.

## 1. Introduction

Myxoma is a rare benign primitive tumor of the mesenchyme [1]; it presents itself like an unpainful, slow-growing mass. It is even more rare at the skeletal muscle level [2] and is named in this case “intramuscular myxoma.” We describe here an exceptional case of intramuscular myxoma in the deltoid, which has the particularity to lyse the surrounding scapular bone. This bone lysis is almost unique; indeed, only 7 cases have been reported since the fifties, but never at the shoulder level.

## 2. Case Presentation

An 83-year-old woman presented with a slow-growing, palpable, painless mass in her left shoulder. She was known for auricular fibrillation, a type of hypothyroidism. The patient had no symptom; she just noticed the apparition of this mass two years earlier. At examination, no limitation in shoulder range motion was found and a mass of about 6 cm diameter was palpable.

**Figure 1:**
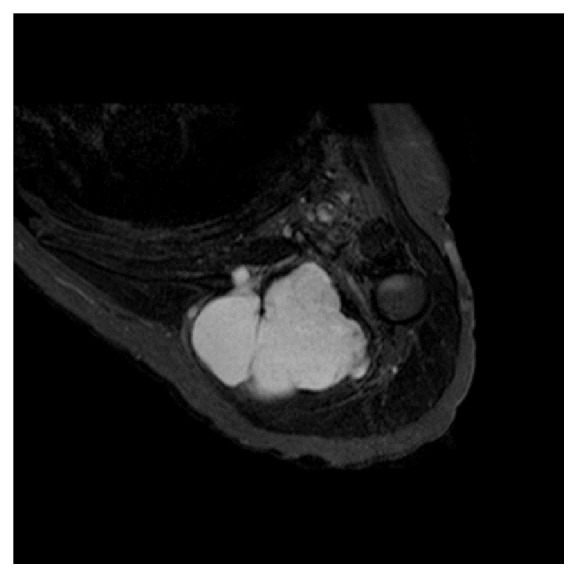
STIR and T2-weighted transverse MRI. Observe the huge cystic-like hyperintense mass growing inside the deltoid muscle and invading the scapula. The mass contains some septations but there is no apparent solid component.

**Figure 2:**
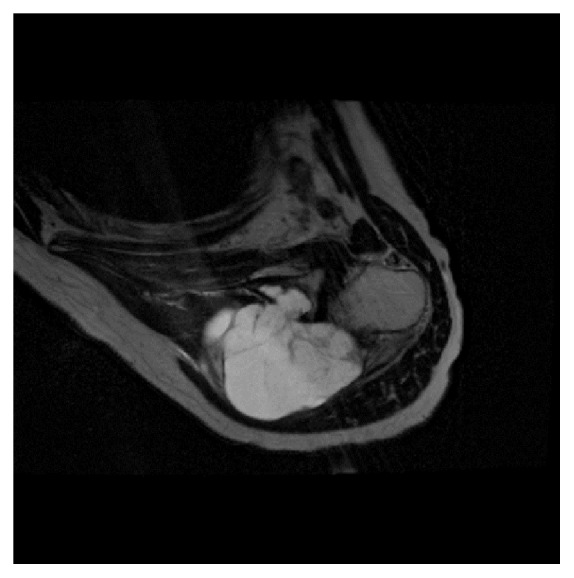
STIR and T2-weighted transverse MRI. Observe the huge cystic-like hyperintense mass growing inside the deltoid muscle and invading the scapula. The mass contains some septations but there is no apparent solid component.

**Figure 3:**
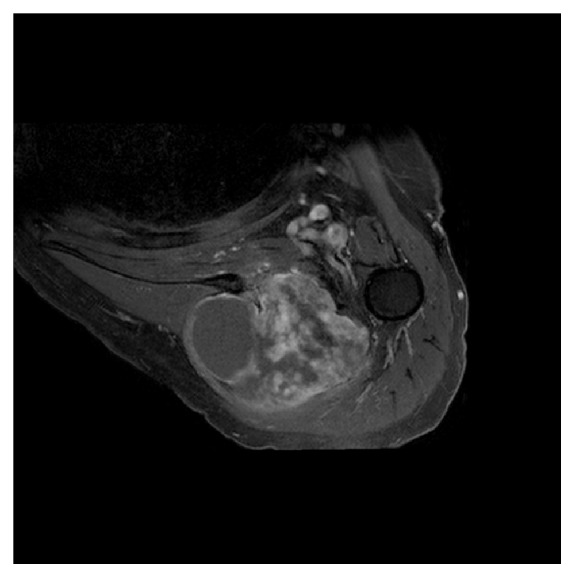
T1 transverse image with fat saturation after intravenous gadolinium injection. Note the enhancement indicating the presence of a solid component inside the mass which in consequence is a pseudocystic mass.

**Figure 4:**
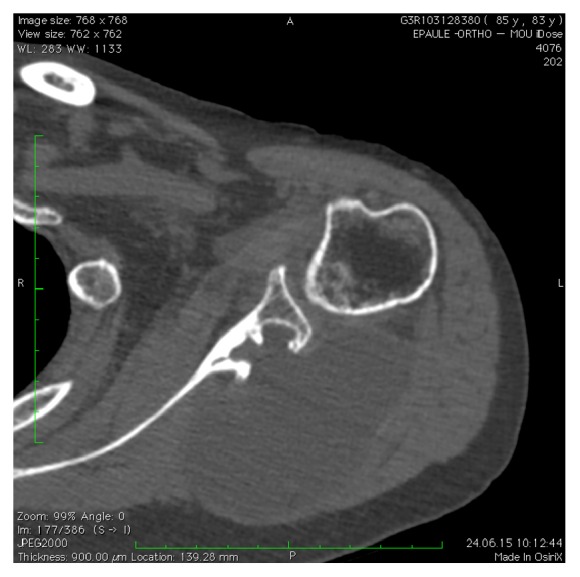
CT scan confirming the invasion of the scapula by the mass.

**Figure 5:**
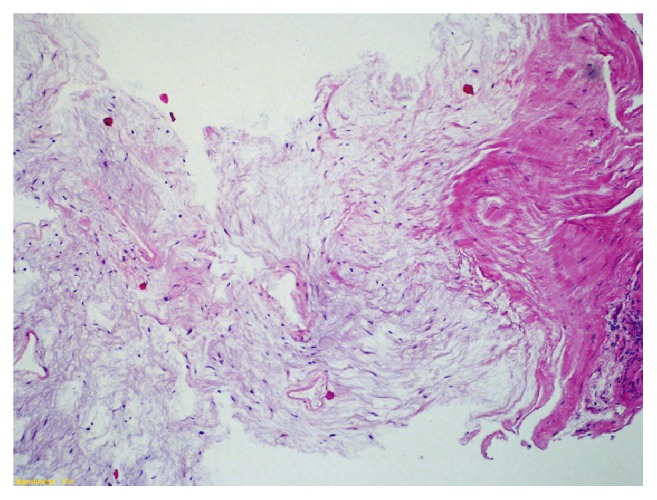
On histological examination, abundant myxoid matrix with few cells is observed. Notice the poor vascularization. Normal adjacent skeletal muscle is present on the left side of Figure 4.

**Figure 6:**
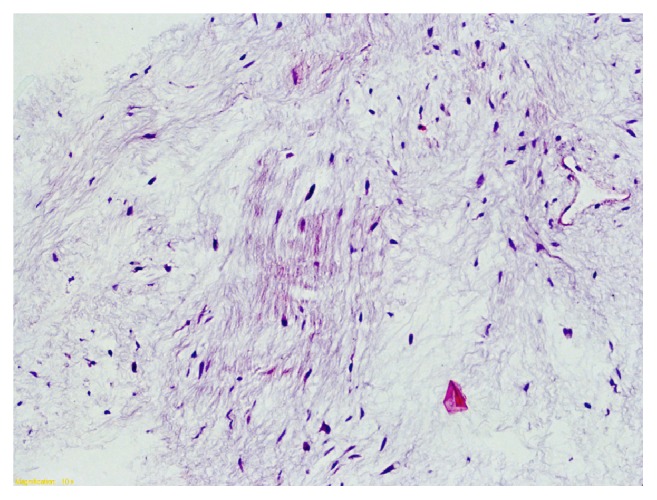
On histological examination, abundant myxoid matrix with few cells is observed. Notice the poor vascularization. Normal adjacent skeletal muscle is present on the left side of Figure 4.

**Figure 7:**
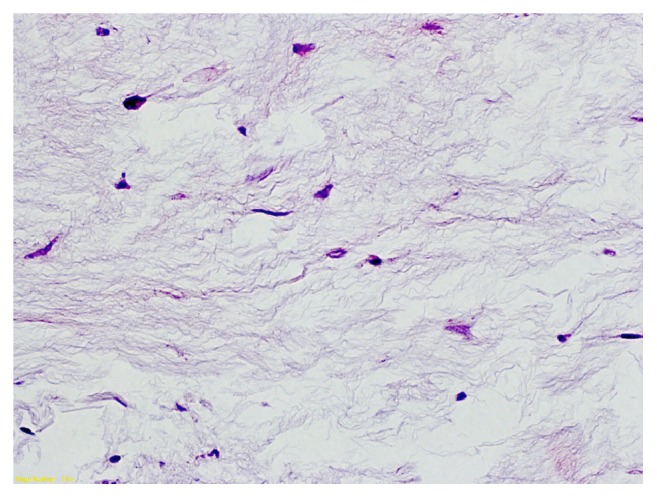
At higher magnification, fusiform cells of little size and regular shape are seen. Elongated aspect nuclei are present, without mitotic activity.

Conventional X-rays were normal. CT scan and magnetic resonance imaging (MRI) were performed and showed an important prescapular necrotic cystic-like mass measuring 9.5 × 6.0 cm (Figures 1–3) with scapular encroachment (bony erosion) (Figure 4). Finally, a guided biopsy under ultrasonographic control was performed. Four samples were taken in the periphery of the lesion. They showed a cystic lesion with necrotic debris in its center. At histological examination, a myxoid aspect with few cells was noticed. Some fusiform cells of little size, regular, elongated aspect nuclei were found, without hyperchromasia or mitotic activity. The myxoid matrix was abundant and loose. The lesion was not vascularized (Figures 5–7). The diagnosis of a benign tumor of myxoma type was retained. Simple follow-up was decided due to the lack of symptoms and the age of the patient. One year later, she was still asymptomatic.

## 3. Discussion

Intramuscular myxomas are localized in skeletal muscles; they represent a distinct subtype of myxomas and have been described for the first time in 1965 by Enzinger [1], constituting only 17% of all soft tissue myxoma cases in his study. They occur more frequently in females and usually affect patients between 40 and 70 years of age [2].

In terms of localization, extracardiac myxomas are rare, and they occur most commonly in the head and skin tissue [3]. Regarding intramuscular myxomas, they have been exceptionally reported in shoulder muscles, thighs, buttocks, or upper extremities [4]. In the present case, the intramuscular myxoma was in the deltoid muscle. Such localization has only been published three times [5–7], but never in conjunction with bone lysis. This bone lysis is in fact very rare, and some odontogenic myxomas with gnathic bone lysis have been described [8] but they presented more aggressive proliferation with cortical lysis and the worst prognostics. To the best of our knowledge, there are only seven cases of extragnathic myxomas associated with bone lysis described in the English literature [9–13].

Histologically, myxoma is a primitive tumor of the mesenchyme composed of undifferentiated stellate cells in a loose mucoid stroma with reticulin fibers; vascularization is poor but focal hypervascularity may be seen and an abundant myxoid matrix is present [14]. The tumor is characterized by the absence of a true capsule but only possessed an incomplete pseudocapsule [8]. These criteria were met in our case (Figures 5–7). The etiology of myxomas remains elusive. Some authors suggested a traumatic origin [2]. It is also possible that growth of polysaccharide-producing cells is implicated in the neoplastic process [1, 15].

Under MRI examination, the myxoma presented a cystic-like aspect partly solid with thick rim enhancement (Figures 1–3) [16]. Usually, intramuscular myxomas appear hypointense on T1-weighted sequences with a characteristic perilesional fat rind and an increased signal in the adjacent muscle on T2-weighted and fluid-sensitive MR sequences can be found [17]. Unfortunately, in our case, these criteria were not all present. But the final diagnosis is always retained on a biopsy, especially to differentiate a simple intramuscular myxoma from a malignant tumor.

The differential diagnosis of intramuscular myxomas includes also aggressive angiomyxoma, myxoid neurofibroma, myxoid liposarcoma, cellular or juxta-articular myxoma, and nodular fasciitis [18, 19]. Because focal areas of hypervascularity and hypercellularity may be present, it is sometimes difficult to differentiate a simple intramuscular myxoma from a malignant tumor. Immunostain for S-100 protein and GNAS 1 mutations can distinguish myxoid liposarcoma and low-grade myxofibrosarcoma from intramuscular myxomas, respectively [20, 21]. In the present case, the diagnosis was clear and additional investigations were not necessary.

Clinically intramuscular myxomas usually present as a painless slow-growing mass; symptoms are due to the compression of surrounding structures [1]. In case of multiple intramuscular myxomas, the Mazabraud syndrome and the McCune-Albright syndrome should be considered, but the first is associated with fibrous dysplasia and the second with polyostotic bone dysplasia, café-au-lait spots, and precocious puberty [22, 23], conditions not present in our patient.

By consensus, the recommended treatment of intramuscular myxomas is surgical excision. However, the recurrence of intramuscular myxomas is rare, restricted to isolated cases, and more commonly associated with syndromes [24–26]. In our case, the decision of a conservative treatment was taken regarding age and lack of symptoms in our patient. One year after the biopsy, the patient was still asymptomatic.

## 4. Conclusion

In the presence of an intramuscular growing mass with associated bone lysis, myxoma as well as malignant neoplasm must be evoked. MRI with gadolinium injection and biopsy should be part of the initial appraisal to obtain a clear diagnosis. Surgical excision is the recommended treatment but every case should be discussed, and conservative treatment with simple follow-up can be an option for this benign tumor.
